# LncRNA LINC01134 Contributes to Radioresistance in Hepatocellular Carcinoma by Regulating DNA Damage Response *via* MAPK Signaling Pathway

**DOI:** 10.3389/fphar.2021.791889

**Published:** 2022-01-31

**Authors:** Zhiyi Wang, Xinxing Wang, Zhonghou Rong, Longfei Dai, Chengkun Qin, Shikang Wang, Wenmao Geng

**Affiliations:** Department of Hepatobiliary Surgery, Shandong Provincial Hospital Affiliated to Shandong First Medical University, Jinan, China

**Keywords:** DNA damage, linc01134, MiR-342-3p, IGF2BP2, MAPK1

## Abstract

Hepatocellular carcinoma (HCC) is a highly mortal cancer that could be treated by radiotherapy. DNA damage response (DDR) is a vital factor affecting cancer development after radiotherapy. Long non-coding RNAs (lncRNAs) have been revealed to regulate DNA damage response and repair in cancer cells. Nevertheless, the function of long intergenic non-protein coding RNA 1134 (LINC01134) has not been explored in DDR. In this study, we targeted digging into the function of LINC01134 in DDR and exploring the underlying mechanism in HCC cells. RT-qPCR was employed to measure LINC01134 expression, and we found LINC01134 was significantly upregulated in HCC cells. Functional analysis suggested that LINC01134 depletion attenuated radioresistance of HCC cells by facilitating DNA damage. *In vivo* assays demonstrated LINC01134 depletion hindered HCC tumor growth. Mechanism assays unveiled LINC01134 sequestered microRNA-342-3p (miR-342-3p) and recruited insulin-like growth factor 2 mRNA binding protein 2 (IGF2BP2) protein to modulate mitogen-activated protein kinase 1 (MAPK1) expression, consequently activating MAPK signaling pathway. Rescue assays validated the LINC01134/miR-342-3p/MAPK1 axis in the radio-resistant HCC cells. In conclusion, LINC01134 might be identified to be a useful biomarker for the therapy of HCC.

## Introduction

Hepatocellular carcinoma (HCC) is a malignancy with high occurrence and mortality (Tang, 2000). It turns out that HCC therapy is facing a great challenge due to multiple pathogenic factors (Gish, 2006). Among all the therapeutic methods of HCC, radiotherapy occupies an important position in improving the survival rate and prognosis of HCC patients (Wang et al., 2018a; Yu and Feng, 2018). However, resistance to radiotherapy, which often results in disease recurrence, decreases the effects of current anticancer treatment for patients with HCC, particularly in the late stages (Bamodu et al., 2020). DNA damage response (DDR), including DNA repair of injured cells, is a cellular response to irradiation, which can maintain cell homeostasis. Molecules that inhibit the expression of proteins in the DDR pathway have been uncovered to improve the impact of radiotherapy (Wu et al., 2020a). Moreover, DNA damage is a crucial factor that influences cancer development and outcome after radiotherapy (Goldstein and Kastan, 2015). Therefore, exploring novel biomarkers and delving into the regulatory mechanism of DNA damage in HCC after radiotherapy is of great significance.

Long non-coding RNAs (lncRNAs) have been discovered to be aberrantly expressed and to affect cancer phenotype in HCC (Lim et al., 2019). Accumulating studies have demonstrated that lncRNAs competitively bind with microRNA (miRNA) as competing endogenous RNAs (ceRNAs) to further modulate mRNA expression, thus influencing cancer development (Zhou et al., 2019). The ceRNA model involving lncRNAs has been extensively discussed in HCC. For instance, Zhong et al. (2020) pointed out that lncRNA SNHG6 influences the prognosis of HCC by targeting miR-17-5p/p62 axis. Dong et al. (2018) revealed that lncRNA SNHG8 accelerates the tumorigenesis and metastasis of HCC by serving as a miR-149-5p sponge. Yang et al. (2019) unraveled that lncRNA CACNA1G-AS1 plays a promoting part in HCC *via* sequestering miR-2392 and modulating C1orf61 expression.

Mounting evidence also suggested that lncRNAs are implicated in radioresistance of HCC cells *via* acting as ceRNAs (Yao et al., 2019). For example, Chen et al. (2018) demonstrated that lncRNA ROR facilitates radioresistance of HCC cells *via* acting as the ceRNA of miR-145 to modulate RAD18 expression. Ma et al. (2018) pointed out that lncRNA H19 affects the radioresistance of HCC cells through regulating miR-193a-3p/PSEN1. Chen et al. revealed that lncRNA NEAT1_2 plays an inhibitory effect on radiosensitivity of HCC cells by regulating the miR-101-3p/WEE1 axis (Chen and Zhang, 2019). LncRNA LINC01134 has been uncovered to be a tumor promoter in HCC (Rong et al., 2020; Zheng et al., 2020). However, the association between LINC01134 and radioresistance or radiosensitivity in HCC has not been explored yet. Hence, the function of LINC01134 in radioresistance of HCC cells along with its ceRNA mechanism is worth investigating.

RNA binding proteins (RBPs) have been reported to modulate the stability and translation of their target mRNAs (Wu et al., 2019). The function of some lncRNAs depends on their interacting proteins, including RBPs (Wang et al., 2020). The interaction of lncRNA, RBP, and mRNA has also been investigated in HCC. For example, lncRNA CASC11 has been confirmed to enhance the stability of E2F1 mRNA *via* recruiting EIF4A3, thereby promoting HCC progression (Song et al., 2020a). LINC00467 has been discovered to prompt HCC cell proliferation and metastasis *via* binding with IGF2BP3 to stabilize TRAF5 mRNA (Jiang et al., 2020). As a result, RBP could also be a valuable subject in this study.

The purpose of the present study is to unveil the impact of LINC01134 on the radioresistance of HCC cells and disclose the latent regulatory mechanism. We hope that this finding could provide more novel potential targets for improving HCC prognosis after radiotherapy.

## Materials and Methods

### Cell Culture

HCC cell lines (Huh7, Hep3B, SNU-182, and SK-HEP-1) and normal epithelial cell line (THLE-3) were involved in this study. Huh7 and Hep3B were provided by Yaji Biotechnology Co., Ltd. (Shanghai, China) and incubated in RPMI-1640 (A4192301, Gibco, Rockville, MD, United States) and minimum essential medium (MEM; A4192101, Gibco), separately. THLE-3, SNU-182, and SK-HEP-1 were procured from American Type Culture Collection (ATCC; Manassas, VA, United States) and cultured in BEGM (CC-3170, Lonza, Basel, Switzerland), RPMI-1640 and Eagle’s Minimum Essential Medium (EMEM; 11095080, Gibco) separately. All media contained 10% fetal bovine serum (FBS; 16140071, Thermo Fisher Scientific, Rockford, IL, United States) and were maintained under a moist environment with 5% CO_2_ at 37°C.

### Quantitative Reverse Transcription PCR (RT-qPCR)

Total RNA extraction was realized using TRIzol Reagent (15596018, Invitrogen, Carlsbad CA, United States). Moreover, PrimeScript™ II Reverse Transcriptase (2690A; TaKaRa, Shiga, Japan) was utilized to obtain cDNA of LINC01134 and MAPK1, following the standard method. The One Step miR cDNA Synthesis Kit (D1801, HaiGene, Harbin, China) was applied to synthesize cDNA of miRNAs, based on the user manual. Quantitative analysis of LINC01134 and MAPK1 was done with SYBR Green PCR Kit (4309155, Applied Biosystems, Foster city, CA, United States) on ABI Prism 7900HT sequence detector (PRISM^®^ 7900HT, Applied Biosystems). For miRNA quantification, HG miRNA SYBR Green PCR Kit (ZY-61500, HaiGene, Harbin, China) was used. Results were analyzed using the 2^−ΔΔCt^ method and standardized to GAPDH or U6. The sequences of primers involved in these assays were listed as follows: LINC01134 (F: TTG​GAC​CAT​GTC​AGT​GAC​GG; R: CAG​AGC​CAG​GTA​GGG​TGT​TG), MAPK1 (F: TCC​TTT​GAG​CCG​TTT​GGA​GG; R: GGT​CAG​CAG​GGC​ATC​ATG​TA), IGF2BP2 (F: GGA​ACA​AGT​CAA​CAC​AGA​CAC​A; R: CGC​AGC​GGG​AAA​TCA​ATC​TG), GAPDH (F: GAC​AGT​CAG​CCG​CAT​CTT​CT; R: GCG​CCC​AAT​ACG​ACC​AAA​TC), U6 (F: TCC​CTT​CGG​GGA​CAT​CCG, R: AAT​TTT​GGA​CCA​TTT​CTC​GAT​TTG​T), miR-140-3p (tac​cac​agg​gta​gaa​cca​cgg), miR-676-3p (ctg​tcc​taa​ggt​tgt​tga​gtt), miR-708-5p (aag​gag​ctt​aca​atc​tag​ctg​gg), miR-28-5p (aag​gag​ctc​aca​gtc​tat​tga​g), miR-1271-5p (ctt​ggc​acc​tag​caa​gca​ctc​a), miR-342-3p (tct​cac​aca​gaa​atc​gca​ccc​gt), miR-2355-3p (att​gtc​ctt​gct​gtt​tgg​aga​t) and miR-6512-3p (ttc​cag​ccc​ttc​taa​tgg​tag​g). The reverse sequence for each miRNA used was “CTC​AAC​TGG​TGT​CGT​GGA”.

### Cell Transfection

The specific short hairpin RNAs (sh-RNAs) to LINC01134 or IGF2BP2 were synthesized by GenePharma (Shanghai, China), and non-specific shRNAs worked as negative control (NC). The pcDNA3.1 vectors (GenePharma) were inserted with MAPK1, and the empty pcDNA3.1 vectors were procured for gene overexpression. MiR-342-3p mimics/inhibitor along with NC mimics/inhibitor was designed by Ribobio (Guangzhou, China). Cell transfection was undertaken for 48 h with Lipofectamine 3000 (L3000075, Invitrogen), as instructed by the manufacturer. Sequences of sh-RNAs and sh-NC used herein were listed as follows: sh-NC (for LINC01134) (5’-CCG​GAA​AGA​TCA​GCA​AAC​ACT​CCG​ACT​CGA​GTC​GGA​GTG​TTT​GCT​GAT​CTT​TTT​TTT​G-3’), sh-LINC01134#1 (5’-CAC​CAT​ACA​ATT​TTA​CTT​TCA​GGC​CCT​CGA​GGG​CCT​GAA​AGT​AAA​ATT​GTA-3’), sh-LINC01134#2 (5’-CAC​CAC​TTC​AAG​TGG​TTT​CTA​GCT​CCT​CGA​GGA​GCT​AGA​AAC​CAC​TTG​AAG-3’), sh-LINC01134#3 (5’-CAC​CGT​GCA​TTT​GCT​GTT​CAT​GTC​CAC​TCG​AGT​GGA​CAT​GAA​CAG​CAA​ATG​CA-3’), sh-NC (for IGF2BP2) (5’-CCG​GTT​CTC​TAA​TTA​TCT​CAG​CAC​ACC​TCG​AGG​TGT​GCT​GAG​ATA​ATT​AGA​GAA​CCG​GTT​TTT​G-3’), sh-IGF2BP2#1 (5’-CAC​CAT​GCA​ATT​CCA​CTT​TAC​CCG​ACT​CGA​GTC​GGG​TAA​AGT​GGA​ATT​GCA-3’) and sh-IGF2BP2#2 (5’-CAC​CAT​GAT​TTC​AAG​AAT​CAT​GCG​GCT​CGA​GCC​GCA​TGA​TTC​TTG​AAA​TCA-3’).

### Cell Counting Kit-8 (CCK-8) Assay

CCK-8 assay was conducted with the use of the CCK-8 Kit. At first, HCC cells were plated in 96-well plates. After cells were cultured for 24, 48, 72, or 96 h, 10 μL of CCK-8 reagent was added into the small wells. Cell viability was measured based on the optical density (OD) value at 450 nm. In the end, a microplate reader was used for OD value analysis.

### Colony Formation Assay

After transfection, SNU-182 and SK-HEP-1 cells were cultured in six-well plates with about 600 cells in each well. After 14 days, colonies were rinsed by PBS and then fixed in methanol for staining with 0.5% crystal violet (V5265, Sigma-Aldrich, St. Louis, MO, United States) solution. Visible clones were manually counted. The experiment was independently performed in triplicate.

### *In Vitro* Irradiation

Cells were inoculated into six-well plates (600 cells/well) and treated with 0, 2, 4, 6, and 8 Gy of irradiation. Next, the irradiated cells were cultured in a complete medium. Two weeks later, surviving colonies were counted manually after being stained with 0.1% crystal violet. The experiment was independently carried out in triplicate.

### Transferase-Mediated dUTP Nick End Labeling Assay

HCC cells fixed by 4% paraformaldehyde (PFA) were treated with TUNEL reagent (12156792910, Roche, Basel, Switzerland) that contained TdT and TMR-dUTP. After cells were stained with DAPI (D9542, Sigma-Aldrich), optical microscopy (DMi1, Leica, Wetzlar, Germany) was used to analyze TUNEL positive cells. The experiment was independently implemented in triplicate.

### Immunofluorescence Staining

Immunofluorescence staining was conducted to examine the formation of gamma histone 2AX (*γ*-H2AX) and 53BP1 foci. In short, cells were seeded in 24-well plates to receive 0 and 4 Gy of irradiation. After 12, 24, or 36 h, the cells were subjected to 4% PFA fixing and 0.1% Triton X-100 (Sigma) permeabilization. Afterwards, the cells were blocked with 1% goat serum (16210072, Gibco), followed by incubation with primary anti-*γ*H2AX (1:100; Abcam) and anti-53BP1 (1:100; Abcam). Then, the cells were co-cultured with secondary antibodies combining with fluorescein isothiocyanate after detached primary antibodies were washed off. Finally, nuclei were counterstained with DAPI. The percentage of *γ*-H2AX or 53BP1-positive cells was analyzed by a fluorescent microscope. The quantification of foci was achieved by Image J software. The experiment was independently conducted in triplicate.

### Comet Assay

DNA damage was assessed by comet assay. The neutral comet assays were conducted with the help of a Reagent Kit for Single Cell Gel Electrophoresis Assay (4250-050-K, Trevigen, United States). The user manual was strictly followed. DNA was stained by 100 μL propidium iodide (PI; 2 μg/ml, HY-D0815, MedChemExpress, NJ, United States) per slide for 30 min in darkness. Photos were captured by the Olympus BX61 capture system (magnification, 20×, Olympus, Tokyo, Japan). Finally, 50–100 cells were analyzed in each group using CaspLab software (CASP 1.2.3 beta 1). This experiment was independently conducted in triplicate.

### Subcellular Fractionation

Subcellular fractionation assay was operated based on the guidance of PARIS™ Kit (AM1921, Invitrogen, Carlsbad, CA, United States). Cell fractionation buffer was used to separate cell cytoplasm. Then, a cell disruption buffer was utilized to acquire cell nuclei. LINC01134 level in two parts was examined by RT-qPCR. U6 and GAPDH were considered as nuclear control or cytoplasmic control, respectively. The experiment was independently performed in triplicate.

### Fluorescent *In Situ* Hybridization and Immunofluorescence (IF)

The two assays were utilized to localize LINC01134 and IGF2BP2 in SNU-182 and SK-HEP-1 cells. At first, 4% PFA was utilized for 15 min fixation at 37°C. After being permeabilized with 0.5% Triton X-100 (R00285, Leagene, Beijing, China), HCC cells were co-cultured with LINC01134 FISH probe in hybridization buffer and then stained with DAPI.

For the IF assay, IGF2BP2 primary antibody was added to incubate with cells at 4°C for a whole night. Then, FITC-conjugated secondary antibody (7076, Cell Signaling Technology, Boston, MA, United States) was added. With the help of a confocal laser microscope (Axio-Imager_LSM-800, Zeiss, Oberkochen, Germany), images were gained. The experiment was independently conducted in triplicate.

### RNA Pull-Down Assay

RNA pull-down assay was completed with the application of Pierce Magnetic RNA-Protein Pull-Down Kit (20164, Thermo Fisher Scientific, Rockford, IL, United States) in light of the provided instruction. SNU-182 and SK-HEP-1 cells were lysed and then cultivated with a biotinylated (Bio)-LINC01134 probe. Bio-NC acted as a control. After magnetic beads (HY-K0208, MedChemExpress, NJ, United States) were added, the precipitated complexes were collected and purified. Finally, relative RNA or protein enrichment was assessed by RT-qPCR or western blot. The experiment was independently performed in triplicate.

### Luciferase Reporter Assay

The fragments of LINC01134 or MAPK1 3’-UTR covering wild-type and mutant-type miR-342-3p binding sites were inserted into the pmirGLO plasmid (Promega, Madison, WI, United States). HCC cells were co-transfected with miR-342-3p mimics or NC mimics along with pmirGLO plasmid for 48 h. Later, the luciferase activity was analyzed by a dual-luciferase reporter assay system (E1910, Promega, Madison, WI, United States). Renilla luciferase activity acted as an internal reference. As for the luciferase activity detection of different signaling pathways, cells were first plated into 96-well plates. Next, the Cignal Finder Reporter Array Kit (336841, QIAGEN, Dusseldorf, Germany) was utilized to measure the luciferase activity of NOTCH pathway, Wnt pathway, Hedgehog pathway, PI3K/AKT pathway, MAPK pathway, and NF-κB pathway, severally. Each experiment was performed in triplicate.

### RNA Binding Protein Immunoprecipitation

RIP assay was done by means of Magna RIP™ RNA-Binding Protein Immunoprecipitation Kit (638970, Merck, Darmstadt, Germany). After cells were lysed in RIP lysis buffer, collected cell lysate was cultivated with the magnetic beads linked to Ago2 antibody (MABE-253, Sigma-Aldrich) or IGF2BP2 antibody. IgG antibody (ab172730, Abcam, Cambridge, MA, United States) served as NC. The enrichment of RNAs was assessed using RT-qPCR. The experiment was independently performed in triplicate.

### Western Blot

Total protein from HCC cells was extracted by RIPA (R0278, Sigma-Aldrich) first. Next, proteins were shifted onto PVDF (IPVH00010, Millipore) membranes after separation on 12% SDS-PAGE (1610174, Bio-Rad Laboratories, Shanghai, China) and then blocked in 5% nonfat milk. The primary antibodies against *γ*H2AX (80312, CST), Cleaved PARP (5625, CST), p-ATM (13050, CST), Rad50 (3427, CST), p-Chk2 (2197, CST), Ku80 (2753, CST), MRE11 (4847, CST), NBS1 (3001, CST), DNA-PKcs (38168, CST), p53 (48818, CST), MAPK1 (4370, CST), ERK (ab32537, Abcam), p-ERK (ab229912, Abcam), JNK (ab76125, Abcam), p-JNK (ab176662, Abcam), p38 (ab170099, Abcam), p-p38 (ab31828, Abcam), HNRNPA1 (ab177152, Abcam), IGF2BP2 (ab124930, Abcam), GAPDH (ab8245, Abcam), and *β*-actin (ab181092, Abcam) were used for incubating cellular protein overnight at 4°C. GAPDH and *β*-actin worked as an internal reference. Secondary antibodies (ab7090, Abcam) were labeled with horseradish peroxidase (HRP) and added for 2 h incubation at room temperature. The western blots were developed by ECL luminous liquid (Pierce, Rockford, IL, United States). The experiment was independently performed in triplicate.

### Xenograft Assay

A total of 24 male BALB/c nude mice (4 weeks old) were bought from the Model Animal Research Center of Nanjing University. All animal experiment procedures were approved by Shandong Provincial Hospital Affiliated to Shandong University. The mice were divided into four groups based on random selection. HCC cells transfected with 0 Gy/sh-NC, 0 Gy/sh-LINC01134#1, 4 Gy/sh-NC, 4 Gy/sh-LINC01134#1, were incubated with Matrigel (1:1), and then 1.0 × 10^5^ HCC cells were subcutaneously inoculated into the mice. When tumors grew to about 8.0 mm in diameter, mice in each group were exposed to radiation. Mice mechanically fixed by a clamp were exposed to 4 Gy radiation at a dose rate of 0.955 Gy/min. Tumor volume was monitored every 5 days. After 30 days, tumors were excised from all sacrificed mice, and tumor weight was measured.

### Statistical Analysis

Experimental results in triplicate were presented as the mean ± standard deviation (SD) and analyzed with the help of GraphPad PRISM 6 (GraphPad, San Diego, CA, United States). Group difference was analyzed by Student’s *t*-test or one-way/two-way analysis of variance (ANOVA), which was regarded to be significant when *p* < 0.05.

## Results

### LINC01134 is Highly Expressed in HCC and LINC01134 Knockdown Impairs Viability of HCC Cells

**FIGURE 1:**
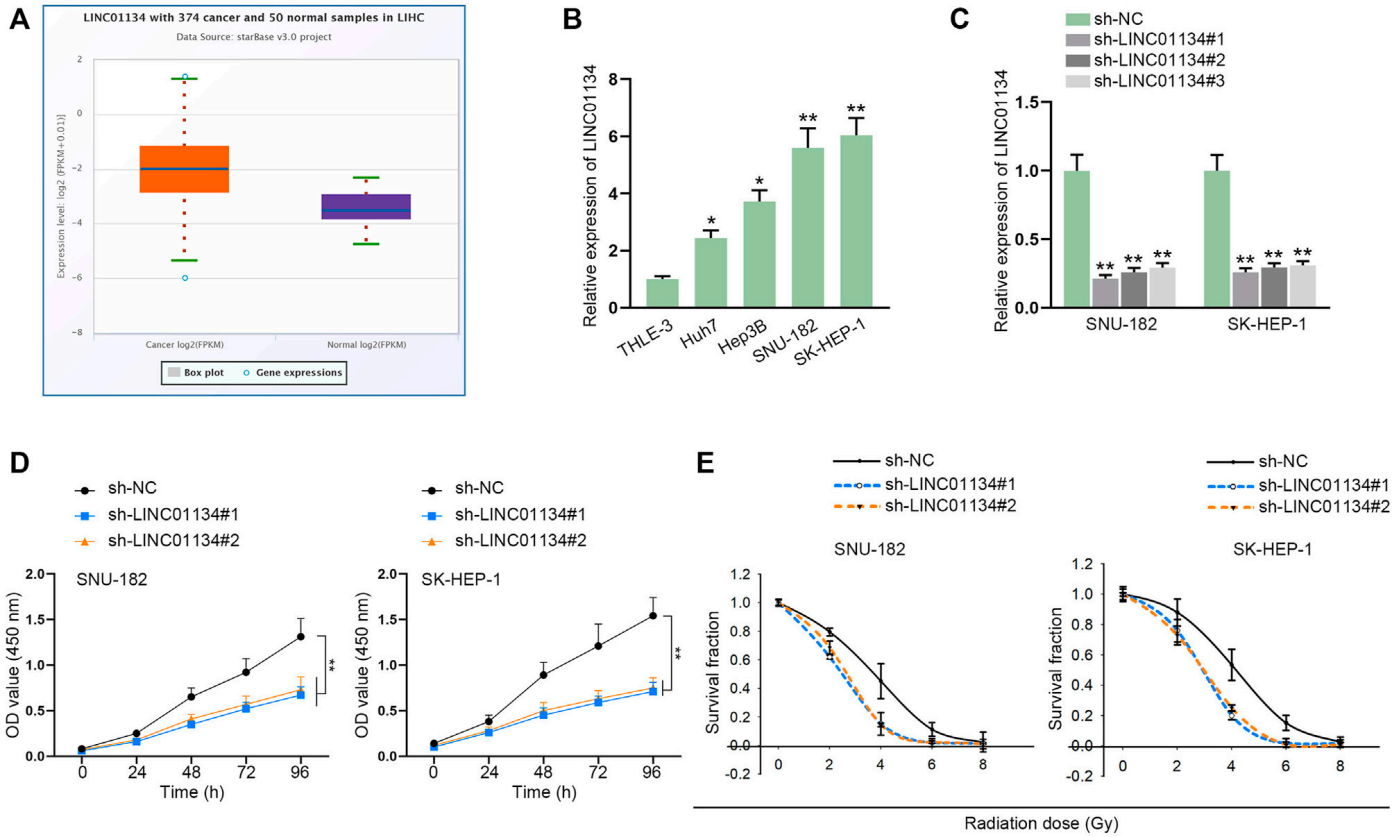
LINC01134 is highly expressed in HCC, and LINC01134 depletion weakens the viability of HCC cells. **(A)** StarBase website was used to search for LINC01134 expression in HCC tumor samples and normal samples. **(B)** RT-qPCR examined LINC01134 expression in HCC cells and normal cells (THLE-3). **(C)** The knockdown efficiency of sh-LINC01134#1/2/3 was evaluated by RT-qPCR in HCC cells. **(D)** The influence of LINC01134 depletion on HCC cell viability was evaluated by a CCK-8 assay. **(E)** With exposure to radiation of 0 Gy, 2 Gy, 4 Gy, 6 Gy, and 8 Gy, colony formation assay was performed to estimate survival fractionation when LINC01134 was inhibited in HCC cells. *p < 0.05, **p < 0.01.

According to starBase (http://starbase.sysu.edu.cn/index.php) prediction, LINC01134 was discovered to be obviously upregulated in liver hepatocellular carcinoma (LIHC) tissues compared with the control group (Figure 1A). RT-qPCR was implemented to examine LINC01134 expression in HCC cell lines (Huh7, Hep3B, SNU-182, and SK-HEP-1) and normal cell line (THLE-3). The result indicated that LINC01134 was significantly upregulated in HCC cells, particularly in SNU-182 and SK-HEP-1 cells (Figure 1B). To cut down the expression of LINC01134, sh-LINC01134#1/2/3 plasmids were transfected into SNU-182 and SK-HEP-1 cells. It turned out sh-LINC01134#1/2/3 efficiently knocked down LINC01134, and sh-LINC01134#1/2 had higher efficiency than sh-LINC01134#3 (Figure 1C). Afterwards, we conducted a CCK-8 assay to assess the changes in cell viability after LINC01134 knockdown. It was found that LINC01134 downregulation weakened HCC cell viability (Figure 1D). Subsequently, data collected from colony formation assay revealed that, in HCC cells with different radiation doses (0, 2, 4, 6, and 8 Gy), knockdown of LINC01134 lowered the survival fraction of HCC cells (Figure 1E). As the survival fraction markedly declined at 4 Gy radiation, this radiation dose was adopted for the following experiments. Overall, LINC01134 expression is higher in LIHC tissues and HCC cells compared to their corresponding control groups, and LINC01134 deficiency led to a decline in HCC cell viability.

### Depletion of LINC01134 Attenuates Radio-Resistance of HCC Cells and Inhibits HCC Tumor Growth

**FIGURE 2:**
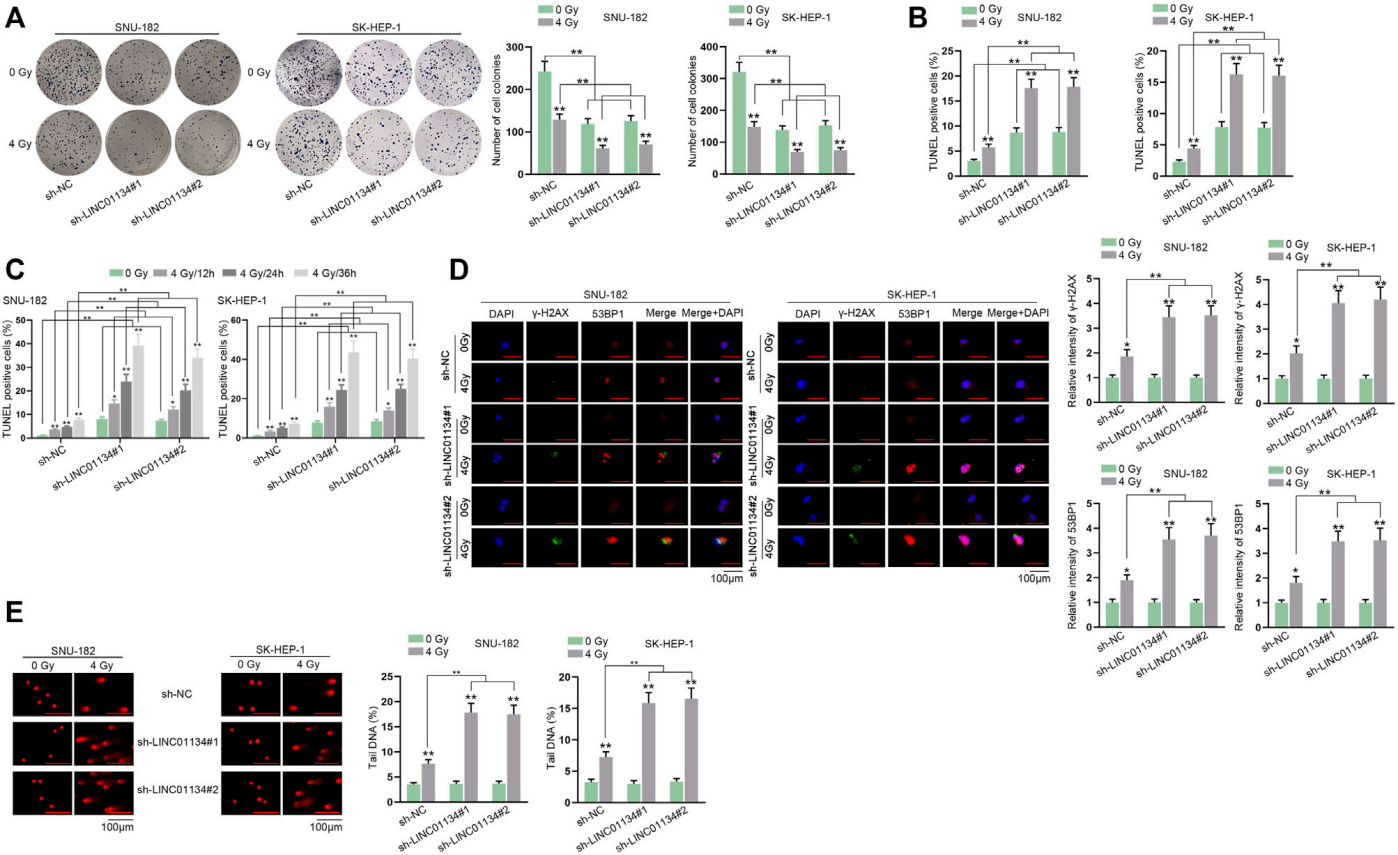
LINC01134 deficiency attenuates radioresistance of HCC by promoting DNA damage. **(A,B)** Colony formation and TUNEL assays were implemented to detect cell proliferation and cell apoptosis under irradiation of 0 Gy and 4 Gy. **(C)** TUNEL assay was carried out in HCC cells exposed to 4 Gy radiation at different time points (12, 24, 36 h). **(D)** Immunofluorescence staining was performed to observe *γ*-H2AX and 53BP1 foci formation (magnification: 100×). **(E)** Comet assay was conducted to examine DNA repair after LINC01134 was downregulated in HCC cells. *p < 0.05, **p < 0.01.

**FIGURE 3:**
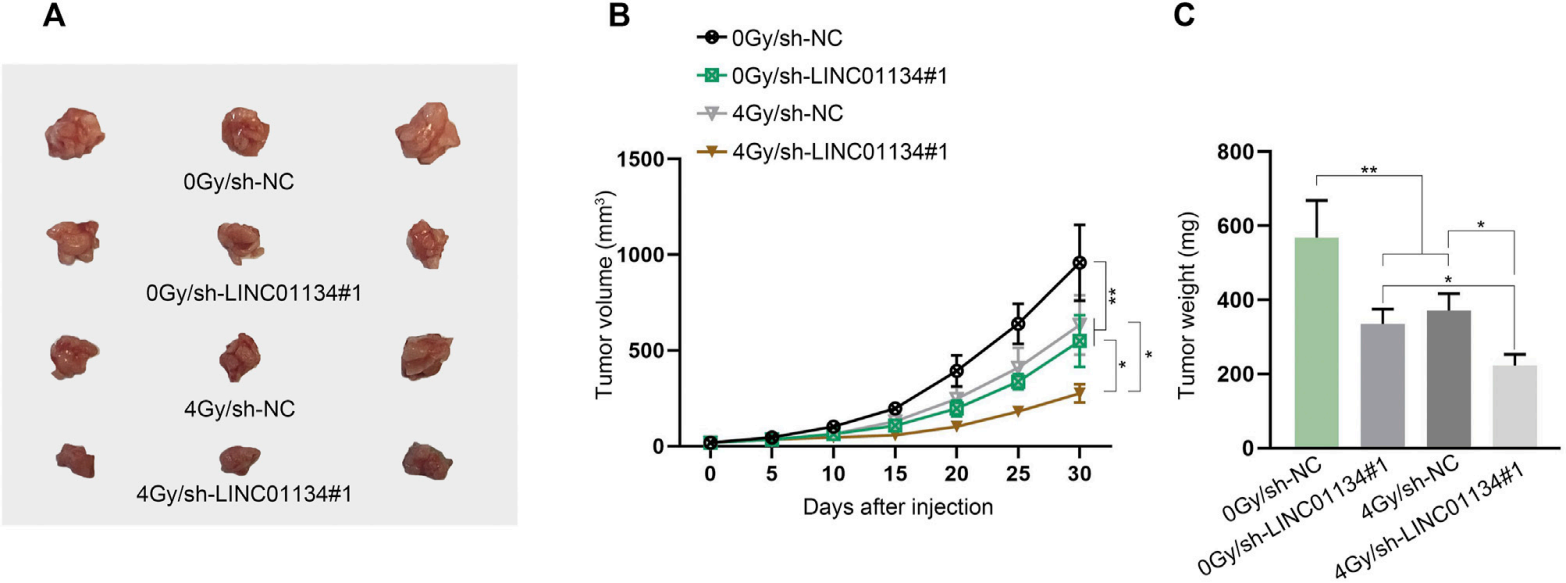
Downregulation of LINC01134 restricts HCC tumor growth. **(A)** Xenograft tumors were observed and photographed after excision. **(B,C)** Tumor volume and weight were monitored to evaluate the tumor growth *in vivo* under different conditions. *p < 0.05, **p < 0.01.

To assess the function of LINC01134 in the radioresistance of HCC cells, functional experiments were implemented. Firstly, LINC01134 expression was examined in HCC cells exposed to 0 Gy or 4 Gy radiation *via* RT-qPCR assay. It turned out that 4 Gy radiation caused no significant change in LINC01134 expression (Supplementary Figure S1A). Then, colony formation assay manifested that 4 Gy radiation dramatically restricted HCC cell proliferation, and LINC01134 downregulation further suppressed cell proliferation (Figure 2A). Conversely, the results of the TUNEL assay disclosed that HCC cell apoptosis facilitated by 4 Gy radiation was further stimulated by LINC01134 depletion (Figure 2B). Moreover, the effects of 4 Gy radiation and LINC01134 knockdown rose with radiation time (Figure 2C). *γ*-H2AX foci are reckoned as a biomarker for DNA damage (Lobachevsky et al., 2020). 53BP1 is a DNA damage response factor (Mirman and de Lange, 2020). Hence, we conducted immunofluorescence staining and found that the formation of *γ*-H2AX and 53BP1 foci increased in cells treated with 4 Gy radiation, and LINC01134 knockdown promoted this trend (Figure 2D). Moreover, the effect of 4 Gy radiation and LINC01134 was the strongest after 12 h radiation and then gradually weakened after 24 and 36 h (Supplementary Figure S1B). Further, the comet assay results also uncovered that after exposure to 4 Gy radiation, the DNA repair capability of SNU-182 and SK-HEP-1 cells was weakened, and LINC01134 inhibition further repressed DNA repair capacity (Figure 2E). Furthermore, xenograft assay was implemented to explore the effects of LINC01134 depletion and 4 Gy radiation *in vivo*. The results showed that tumor growth was highly restricted after exposure to 4 Gy radiation. Under 4 Gy radiation, downregulated LINC01134 further hindered tumor growth (Figures 3A–C; Supplementary File S1). Taken together, silencing of LINC01134 restricts the radioresistance in HCC and impedes HCC tumor growth.

### LINC01134 Serves as a Sponge for miR-342-3p

**FIGURE 4:**
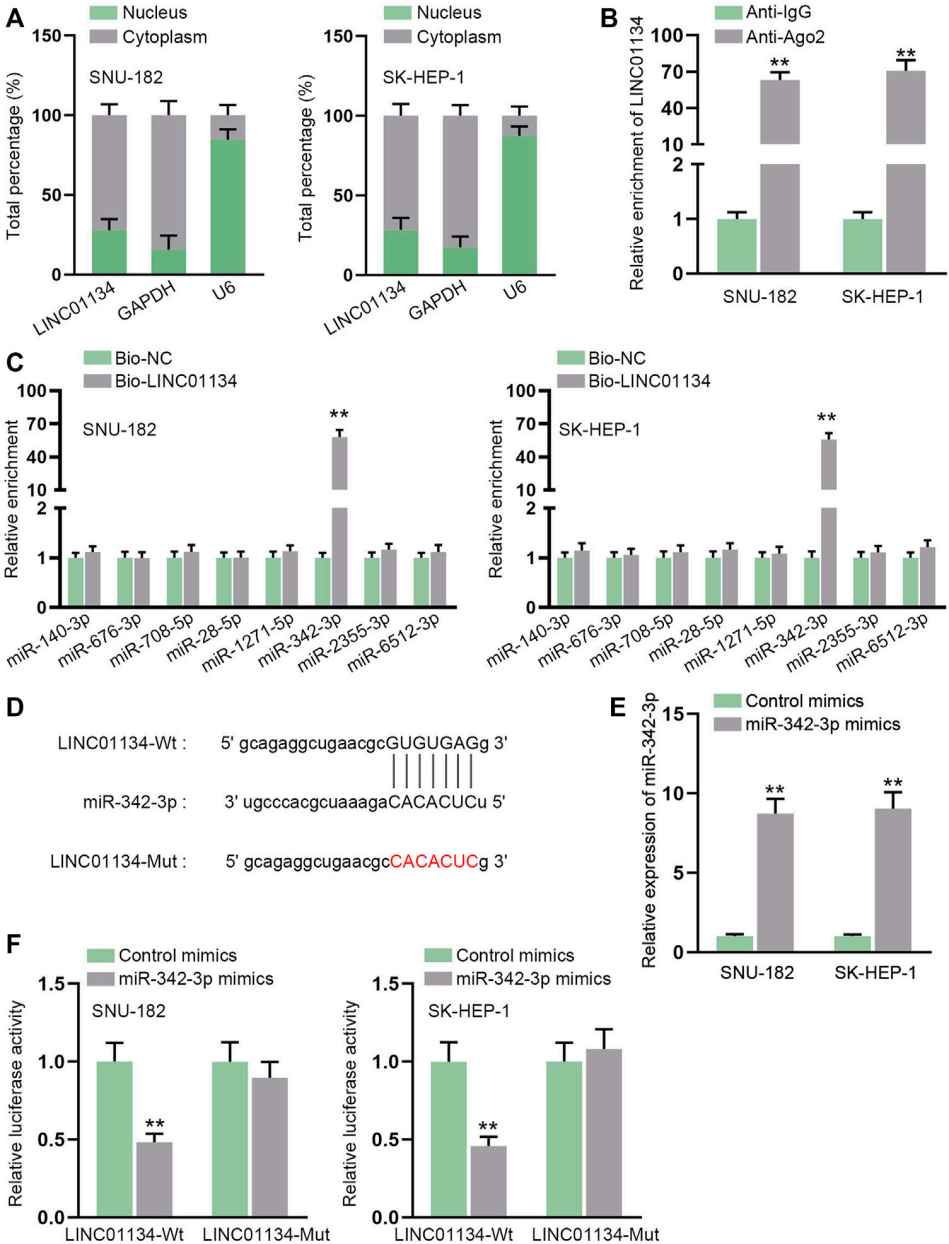
LINC01134 serves as a sponge for miR-342-3p. **(A)** Subcellular fractionation assay was implemented to ascertain the location of LINC01134 in HCC cells. **(B)** RIP assay was done to verify the interaction between LINC01134 and Ago2 protein. **(C)** RNA pull-down assay was employed to detect the enrichment of predicted eight miRNAs in the biotin-labeled LINC01134 probe. **(D)** StarBase was employed to project the binding sequence between LINC01134 and miR-342-3p. **(E)** The overexpression efficiency of miR-342-3p mimics was examined by RT-qPCR in HCC cells. **(F)** Luciferase reporter assay was operated to test the luciferase activity in the LINC01134-Wt group and LINC01134-Mut group after transfection of miR-342-3p mimics. **p < 0.01.

Through subcellular fractionation assay, we observed that LINC01134 was prominently localized in HCC cell cytoplasm (Figure 4A), which indicated that LINC01134 might be involved in post-transcriptional events. RIP assay demonstrated that LINC01134 could be detected in the Ago2-precipitated complex, suggesting that LINC01134 might function as a ceRNA by interacting with miRNAs (Figure 4B). With the application of starBase, eight possible miRNAs were predicted on the condition of Pan-Cancer ≥ 4. Data from the RNA pull-down assay proved that only miR-342-3p had a strong affinity with LINC01134 (Figure 4C). The alignment between LINC01134 and miR-342-3p was exhibited in Figure 4D. After transfection of miR-342-3p mimics, miR-342-3p was overexpressed in SNU-182 and SK-HEP-1 cells (Figure 4E). Subsequently, the luciferase reporter assay attested that the luciferase activity in the LINC01134-Wt group rather than the LINC01134-Mut group was decreased after transfection of miR-342-3p mimics (Figure 4F). To conclude, miR-342-3p is sponged by LINC01134 in HCC cells.

### LINC01134 Sponges miR-342-3p and Modulates MAPK1 Expression to Activate MAPK Signaling Pathway

**FIGURE 5:**
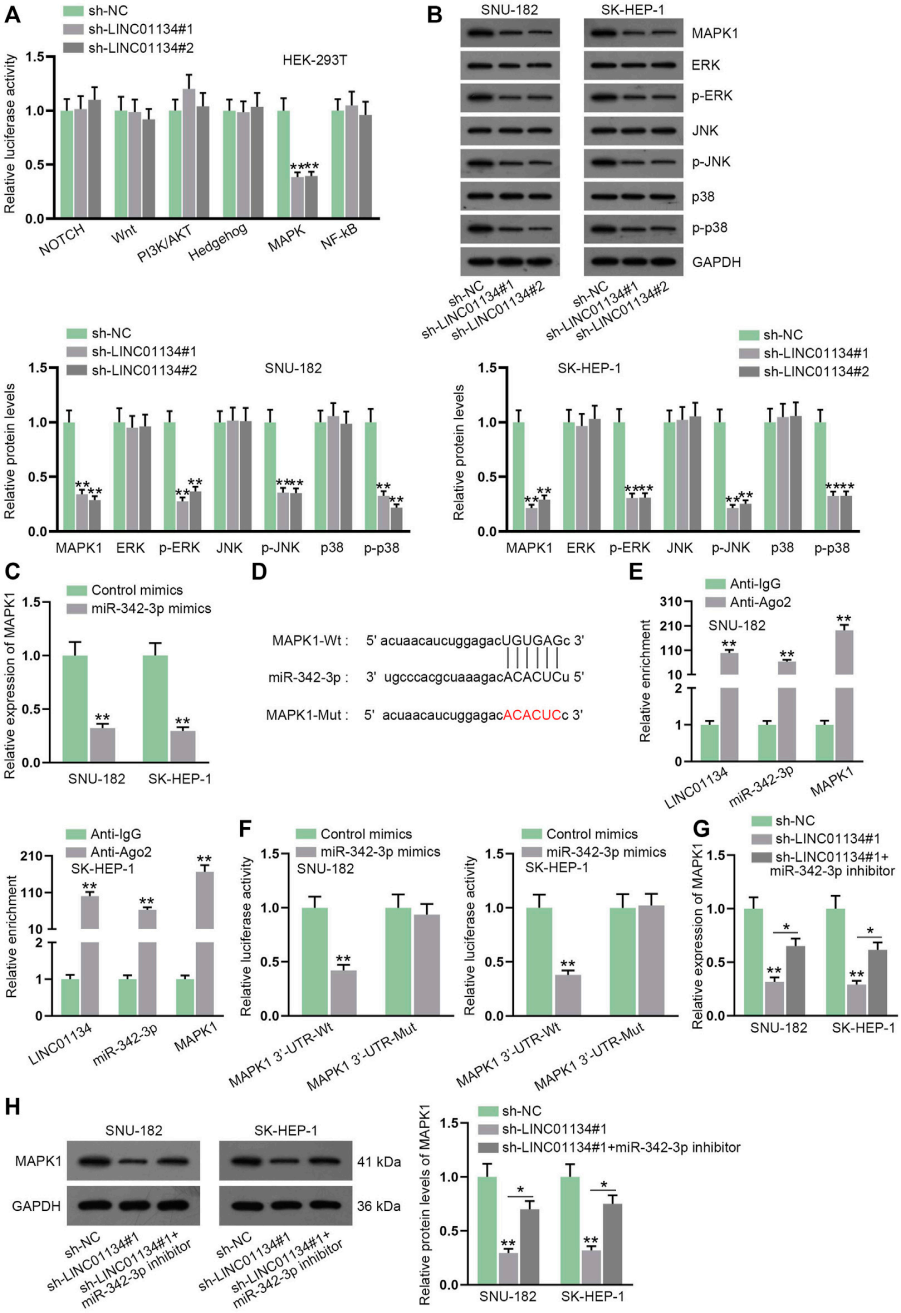
LINC01134 sponges miR-342-3p and modulates MAPK1 expression to activate MAPK signaling pathway. **(A)** Luciferase reporter assay was done to analyze the luciferase activity of six common signaling pathways when LINC01134 was downregulated. **(B)** Western blot was conducted to analyze the protein levels of MAPK1 and downstream factors of the MAPK signaling pathway. **(C)** RT-qPCR was executed to detect MAPK1 expression after miR-342-3p was upregulated. **(D)** The wild-type and mutated binding regions between miR-342-3p and MAPK1 were displayed. **(E)** RIP assay was performed to evaluate the enrichment of LINC01134, miR-342-3p, and MAPK1 in Anti-Ago2. **(F)** Luciferase reporter assay was employed to test the binding of miR-342-3p and MAPK1. **(G,H)** MAPK1 expression was examined in the sh-NC group, sh-LINC01134#1 group, and sh-LINC01134#1+miR-342-3p inhibitor group by RT-qPCR and western blot assays. *p < 0.05, **p < 0.01.

Given the experimental results of the luciferase reporter assay, we uncovered that depletion of LINC01134 lessened the luciferase activity of the MAPK signaling pathway (Figure 5A). In addition, we found that MAPK1 (also known as ERK2), a crucial regulator in the MAPK signaling pathway (Reyes-Gibby et al., 2016), was also a potential target gene of miR-342-3p. Accordingly, we conducted a western blot to analyze whether LINC01134 deficiency influenced MAPK1 and downstream factors of the MAPK signaling pathway. The results manifested that the protein levels of MAPK1, p-ERK (also referring to p-ERK1), p-JNK, and p-p38 were all cut down after LINC01134 was knocked down (Figure 5B; Supplementary File S2). Outcomes of RT-qPCR also demonstrated that miR-342-3p upregulation resulted in the decline of MAPK1 expression (Figure 5C). The projected binding region between miR-342-3p and MAPK1 was demonstrated in Figure 5D. Moreover, the RIP assay showed that the LINC01134, miR-342-3p, and MAPK1 were all enriched in the Ago2 groups (Figure 5E). The binding relation between miR-342-3p and MAPK1 was then validated by luciferase reporter assay as the luciferase activity of MAPK1 3’-UTR-Wt declined due to miR-342-3p overexpression (Figure 5F). Moreover, the decrease in MAPK1 expression on account of LINC01134 interference was partially restored by a miR-342-3p inhibitor (Figures 5G,H; Supplementary File S2). To summarize, LINC01134 modulates MAPK1 expression *via* sponging miR-342-3p to activate MAPK signaling pathway.

### LINC01134 Recruits IGF2BP2 Protein to Stabilize MAPK1 mRNA

**FIGURE 6:**
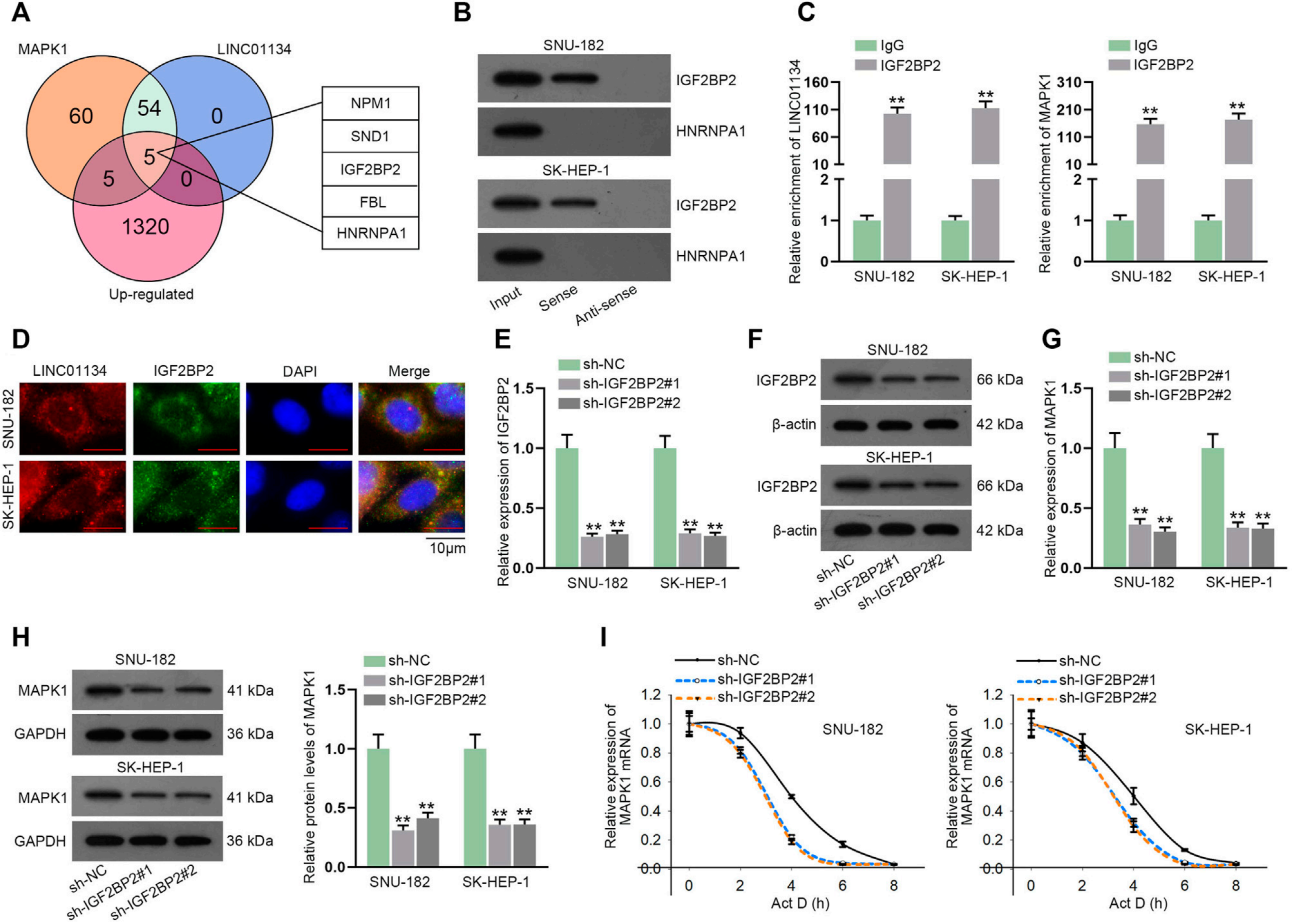
LINC01134 recruits IGF2BP2 protein to stabilize MAPK1 mRNA. **(A)** The overlap of the Venn diagram demonstrated five common potential RBPs of LINC01134 and MAPK1. **(B)** RNA pull-down assay was operated to check the affinity of LINC01134 with IGF2BP2 or HNRNPA1. **(C)** RIP assay was implemented to testify the binding between LINC01134/MAPK1 and IGF2BP2. **(D)** FISH and IF analysis was done to indicate the co-localization of LINC01134 and IGF2BP2 in the cytoplasm of HCC cells (magnification: 1000×). **(E,F)** IGF2BP2 expression was detected through RT-qPCR and western blot assays in SNU-182 and SK-HEP-1 cells upon IGF2BP2 depletion. **(G,H)** MAPK1 expression was tested after IGF2BP2 was silenced. **(I)** The stability of MAPK1 mRNA was checked by RT-qPCR when IGF2BP2 was downregulated. **p < 0.01.

Based on the previous findings that miR-342-3p inhibitor could not entirely counteract the effect of LINC01134 knockdown on MAPK1 expression, we speculated there might exist other potential regulatory mechanisms for LINC01134 to regulate MAPK1. Venn diagram exhibited five candidate RBPs (NPM1, SND1, IGF2BP2, FBL, and HNRNPA1) screened out by starBase and GEPIA database (http://gepia.cancer-pku.cn/index.html) (Figure 6A). Given that HNRNPA1 and IGF2BP2 have been widely discovered to be able to regulate the progression of various cancers, including HCC (Simon et al., 2014; Wen et al., 2020), these two candidate RBPs were selected to engage in the following experiments. RNA pull-down assay uncovered that IGF2BP2 rather than HNRNPA1 was pulled down by biotin-labeled LINC01134 probe (Figure 6B; Supplementary File S2). The binding between LINC01134/MAPK1 and IGF2BP2 was further testified by the RIP assay (Figure 6C). FISH and IF analysis further disclosed the co-localization of LINC01134 and IGF2BP2 in HCC cell cytoplasm (Figure 6D). Next, the knockdown efficacy of sh-IGF2BP2#1/2 was confirmed to be high by RT-qPCR and western blot (Figures 6E,F; Supplementary File S2). From the data of RT-qPCR and western blot detection, MAPK1 expression at mRNA and protein levels was decreased when IGF2BP2 was downregulated (Figures 6G,H; Supplementary File S2). Moreover, the stability of MAPK1 mRNA in the HCC cells treated with actinomycin D (Act D) was lowered by IGF2BP2 inhibition (Figure 6I). In conclusion, LINC01134 interacts with IGF2BP2 protein to stabilize MAPK1 mRNA.

### LINC01134 Participates in the Regulation of HCC Cell Radioresistance by Enhancing MAPK1 Expression

**FIGURE 7:**
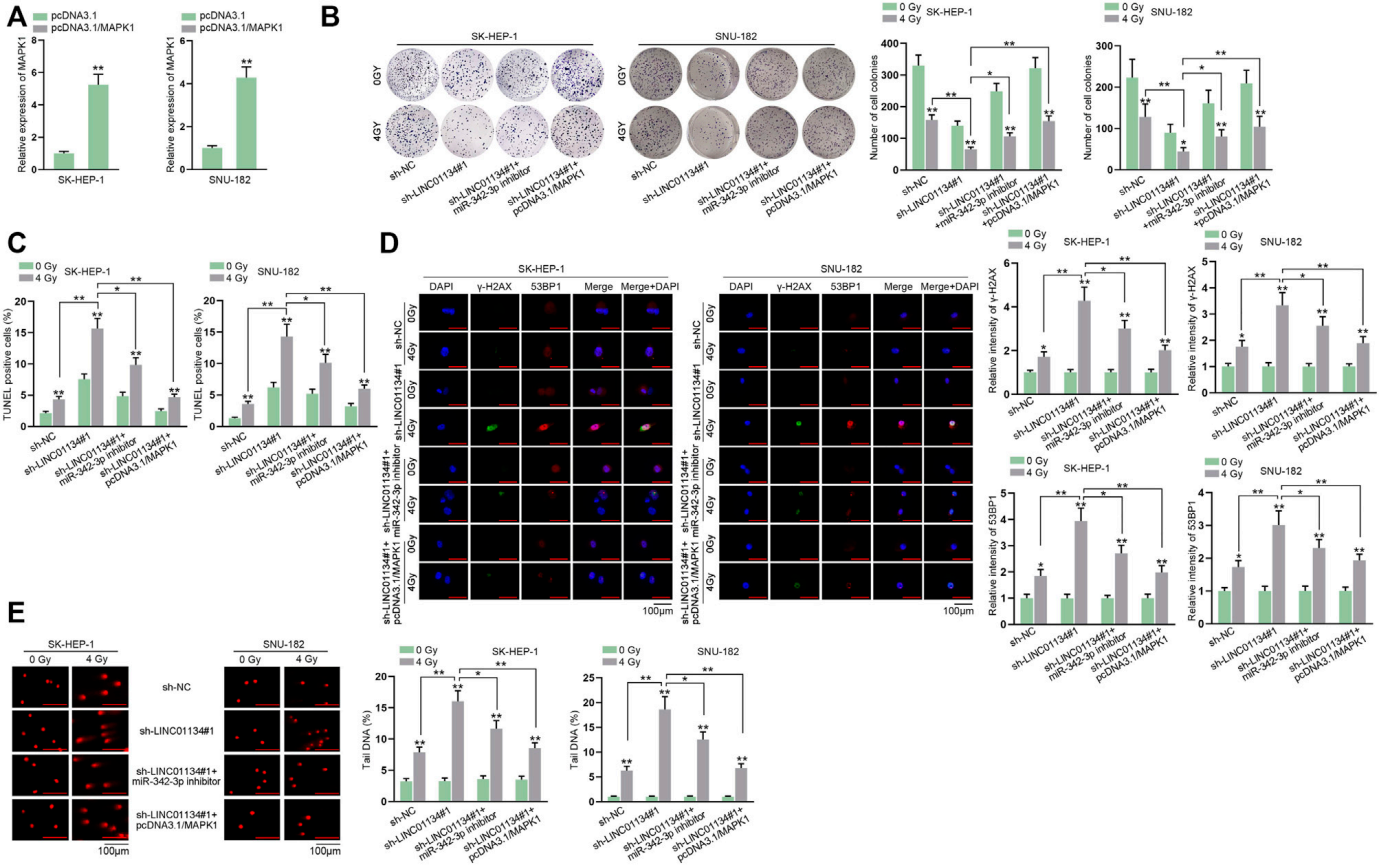
LINC01134 participates in the regulation of radioresistance of HCC cells by enhancing MAPK1 expression. **(A)** The overexpression efficacy of pcDNA3.1-MAPK1 was tested by RT-qPCR in HCC cells. HCC cells were transfected with different plasmids: sh-NC, sh-LINC01134#1, sh-LINC01134#1+miR-342-3p inhibitor, and sh-LINC01134#1+pcDNA3.1/MAPK1. **(B,C)** Cell proliferation and cell apoptosis were observed by colony formation and TUNEL assays. **(D)** The formation of *γ*-H2AX and 53BP1 foci was assessed in HCC cells respectively transfected with indicated plasmids (magnification: 100×). **(E)** Comet assay was performed to evaluate DNA repair under different conditions. *p < 0.05, **p < 0.01.

Before implementing rescue assays, MAPK1 was upregulated by transfection of pcDNA3.1/MAPK1 into HCC cells (Figure 7A). Consequences of colony formation assay illustrated that 4 Gy radiation hampered cell proliferation, and LINC01134 downregulation further restrained cell proliferation. At the same time, this effect was partially restored by miR-342-3p inhibitor and completely restored by MAPK1 overexpression (Figure 7B). The increased apoptotic cells due to 4 Gy radiation were further elevated by LINC01134 depletion, which was partially recovered by miR-342-3p downregulation while being completely recovered by MAPK1 upregulation (Figure 7C). Similarly, the suppressive effect of 4 Gy radiation on the formation of *γ*-H2AX and 53BP1 foci was enhanced by the LINC01134 knockdown, which was partially offset by miR-342-3p inhibition and completely counteracted by upregulation of MAPK1 (Figure 7D). The comet assay results also suggested that, under the conditions of 4 Gy radiation, the limited DNA repair capacity induced by LINC01134 deficiency was partially rescued by miR-342-3p inhibitor and completely rescued by overexpression of MAPK1 (Figure 7E). To sum up, the inhibiting effect of LINC01134 interference on the radioresistance of HCC cells is partially abrogated by miR-342-3p downregulation and fully abolished by MAPK1 upregulation.

## Discussion

HCC is a common mortal cancer worldwide (Chen et al., 2020). Radiotherapy has been verified to be effective for treating HCC (Rim et al., 2021). However, abnormality of DDR, which includes DNA repair, results in radioresistance and restrains radiotherapy effectiveness in HCC patients (Sun et al., 2020). It has been uncovered that lncRNAs play an indispensable part in the regulation of the DNA damage/repair network (Arjumand et al., 2018; Su et al., 2018). For instance, lncRNA NEAT1 leads to DNA damage in multiple myeloma (Taiana et al., 2020). LncRNA LINP1 enhances DNA repair in triple-negative breast cancer (Zhang et al., 2016). LncRNA PVT1 regulates DNA repair in nasopharyngeal carcinoma (He et al., 2018). Our study revealed that LINC01134 was highly expressed in HCC and was connected with the survival fraction of HCC cells upon exposure to radiation. It was also found that knockdown of LINC01134 reduced the radioresistance of HCC cells *via* promoting DNA damage and inhibiting DNA repair. Moreover, we noticed LINC01134 reduction hampered HCC tumor growth *via in vivo* assays. A former study has proved LINC01134 displays a high expression in HCC with oxaliplatin (OXA) resistance, and higher LINC01134 expression indicates poorer OXA therapeutic efficacy (Ma et al., 2021). Consistent with this study, we also discovered that LINC01134 displayed a high expression level in HCC, and LINC01134 could enhance the radioresistance of HCC cells.

CeRNA mechanism has been disclosed to involve in the pathogenesis of HCC (Long et al., 2019). For instance, lncRNA MIAT contributes to proliferative and invasive abilities of HCC cells through sponging miR-214 (Huang et al., 2018). LncRNA FAL1 prompts cell malignant behaviors *via* functioning as a ceRNA of miR-1236 in HCC cells (Li et al., 2018). Through the isolation of cytoplasmic and nuclear RNA, we found that LINC01134 might function as a ceRNA for it was chiefly accumulated in the cytoplasm of HCC cells. Afterwards, miR-342-3p was chosen based on starBase prediction and results of RNA pull-down assay. The following mechanism assays confirmed the binding affinity between miR-342-3p and LINC01134. MiR-342-3p has been widely studied in HCC and determined to be a tumor suppressor in HCC (Gao et al., 2017; Fan et al., 2018). Consistent with these findings, we found that miR-342-3p downregulation rescued the decreased radio-resistance of HCC cells caused by LINC01134 deficiency. The lncRNA-miRNA-mRNA ceRNA network has been identified to play pivotal parts in multiple tumors (Wu et al., 2020b). In the same way, we found that MAPK1 was a target gene of miR-342-3p. MiR-342-3p knockdown could restore the decline of MAPK1 expression caused by LINC01134 reduction. Furthermore, MAPK1 augment completely recovered the repressed radioresistance of HCC cells on account of LINC01134 downregulation. MAPK signaling pathway is responsible for the initiation and promotion of HCC (Dimri and Satyanarayana, 2020). The activation of the MAPK pathway has also been correlated with DDR (Rezatabar et al., 2019). As an important part of the MAPK signaling pathway, MAPK1 has been illustrated to connect to the development of HCC (Wang et al., 2018b; Ye et al., 2020). In line with the abovementioned research, our study also validated that MAPK1 affected HCC cell growth *via* modulating DDR.

Furthermore, RNA binding protein (RBP) network has been viewed as vital factors in human diseases, and it has been uncovered that lncRNAs could bind with RBPs to exert their functions (Brinegar and Cooper, 2016; Song et al., 2020b). For instance, lncRNA CERS6-AS1 plays a cancer-promoting role in breast cancer *via* recruiting IGF2BP3 to strengthen the stability of CERS6 mRNA (Bao et al., 2020). According to starBase and experiment results, IGF2BP2 was uncovered to be the shared RBP of LINC01134 and MAPK1. IGF2BP2 has been identified to be implicated in the malignant phenotype of cancer cells (Cao et al., 2018). In addition, IGF2BP2 can stabilize mRNA (Huang et al., 2019). Likewise, this study discovered that IGF2BP2 was recruited by LINC01134 to stabilize MAPK1 mRNA.

In summary, LINC01134 is distinctly upregulated in HCC, and LINC01134 depletion reduces the radioresistance of HCC cells *via* inducing DNA damage. From the perspective of mechanism, LINC01134 enhances MAPK1 expression *via* binding with miR-342-3p and IGF2BP2 protein, by which it is involved in the radioresistance of HCC cells *via* activation of the MAPK signaling pathway. All these findings suggest that LINC01134 might act as a potential target for enhancing the radiotherapy effect of HCC.

## Data Availability

The original contributions presented in the study are included in the article/Supplementary Material, further inquiries can be directed to the corresponding author.

## Supplementary Material

Supplementary Figure S1
**(A)** LINC01134 expression was detected in HCC cells treated with 4 Gy radiation by RT-qPCR assay. **(B)** Immunofluorescence staining assay was performed to measure the intensity of *γ*-H2AX after LINC01134 knockdown under the conditions of 4 Gy/12 h, 4 Gy/24 h, and 4 Gy/36 h. **p* < 0.05, ***p* < 0.01.

Supplementary File S1 Uncropped pictures for Figure 3A.

Supplementary File S2 Original protein blots for Figures 5B,H, 6B,F,H.

## References

[B1] Arjumand W. Asiaf A. Ahmad S. T. (2018). Noncoding RNAs in DNA Damage Response: Opportunities for Cancer Therapeutics. Methods Mol. Biol. 1699, 3–21. 10.1007/978-1-4939-7435-1_1 29086365

[B2] Bamodu O. A. Chang H. L. Ong J. R. Lee W. H. Yeh C. T. Tsai J. T. (2020). Elevated PDK1 Expression Drives PI3K/AKT/MTOR Signaling Promotes Radiation-Resistant and Dedifferentiated Phenotype of Hepatocellular Carcinoma. Cells 9 (3). 10.3390/cells9030746 PMC7140693 32197467

[B3] Bao G. Huang J. Pan W. Li X. Zhou T. (2020). Long Noncoding RNA CERS6-AS1 Functions as a Malignancy Promoter in Breast Cancer by Binding to IGF2BP3 to Enhance the Stability of CERS6 mRNA. Cancer Med. 9 (1), 278–289. 10.1002/cam4.2675 31701672 PMC6943159

[B4] Brinegar A. E. Cooper T. A. (2016). Roles for RNA-Binding Proteins in Development and Disease. Brain Res. 1647, 1–8. 10.1016/j.brainres.2016.02.050 26972534 PMC5003702

[B5] Cao J. Mu Q. Huang H. (2018). The Roles of Insulin-like Growth Factor 2 mRNA-Binding Protein 2 in Cancer and Cancer Stem Cells. Stem Cell Int 2018, 4217259. 10.1155/2018/4217259 PMC5874980 29736175

[B6] Chen X. Zhang N. (2019). Downregulation of lncRNA NEAT1_2 Radiosensitizes Hepatocellular Carcinoma Cells through Regulation of miR-101-3p/WEE1 axis. Cell Biol Int 43 (1), 44–55. 10.1002/cbin.11077 30488993

[B7] Chen Y. Shen Z. Zhi Y. Zhou H. Zhang K. Wang T. (2018). Long Non-coding RNA ROR Promotes Radioresistance in Hepatocelluar Carcinoma Cells by Acting as a ceRNA for microRNA-145 to Regulate RAD18 Expression. Arch. Biochem. Biophys. 645, 117–125. 10.1016/j.abb.2018.03.018 29559320

[B8] Chen Z. Xie H. Hu M. Huang T. Hu Y. Sang N. (2020). Recent Progress in Treatment of Hepatocellular Carcinoma. Am. J. Cancer Res. 10 (9), 2993–3036. 33042631 PMC7539784

[B9] Dimri M. Satyanarayana A. (2020). Molecular Signaling Pathways and Therapeutic Targets in Hepatocellular Carcinoma. Cancers (Basel) 12 (2), 491. 10.3390/cancers12020491 PMC7072513 32093152

[B10] Dong J. Teng F. Guo W. Yang J. Ding G. Fu Z. (2018). lncRNA SNHG8 Promotes the Tumorigenesis and Metastasis by Sponging miR-149-5p and Predicts Tumor Recurrence in Hepatocellular Carcinoma. Cell Physiol Biochem 51 (5), 2262–2274. 10.1159/000495871 30537734

[B11] Fan H. Lv P. Mu T. Zhao X. Liu Y. Feng Y. (2018). LncRNA n335586/miR-924/CKMT1A axis Contributes to Cell Migration and Invasion in Hepatocellular Carcinoma Cells. Cancer Lett. 429, 89–99. 10.1016/j.canlet.2018.05.010 29753758

[B12] Gao Y. Zhang S. G. Wang Z. H. Liao J. C. (2017). Down-regulation of miR-342-3p in Hepatocellular Carcinoma Tissues and its Prognostic Significance. Eur. Rev. Med. Pharmacol. Sci. 21 (9), 2098–2102. 28537676

[B13] Gish R. G. (2006). Hepatocellular Carcinoma: Overcoming Challenges in Disease Management. Clin. Gastroenterol. Hepatol. 4 (3), 252–261. 10.1016/j.cgh.2006.01.001 16527686

[B14] Goldstein M. Kastan M. B. (2015). The DNA Damage Response: Implications for Tumor Responses to Radiation and Chemotherapy. Annu. Rev. Med. 66, 129–143. 10.1146/annurev-med-081313-121208 25423595

[B15] He Y. Jing Y. Wei F. Tang Y. Yang L. Luo J. (2018). Long Non-coding RNA PVT1 Predicts Poor Prognosis and Induces Radioresistance by Regulating DNA Repair and Cell Apoptosis in Nasopharyngeal Carcinoma. Cell Death Dis 9 (2), 235. 10.1038/s41419-018-0265-y 29445147 PMC5833381

[B16] Huang S. Wu Z. Cheng Y. Wei W. Hao L. (2019). Insulin-like Growth Factor 2 mRNA Binding Protein 2 Promotes Aerobic Glycolysis and Cell Proliferation in Pancreatic Ductal Adenocarcinoma *via* Stabilizing GLUT1 mRNA. Acta Biochim. Biophys. Sin (Shanghai) 51 (7), 743–752. 10.1093/abbs/gmz048 31089713

[B17] Huang X. Gao Y. Qin J. Lu S. (2018). lncRNA MIAT Promotes Proliferation and Invasion of HCC Cells *via* Sponging miR-214. Am. J. Physiol. Gastrointest. Liver Physiol. 314 (5), G559–g65. 10.1152/ajpgi.00242.2017 29097358

[B18] Jiang W. Cheng X. Wang T. Song X. Zheng Y. Wang L. (2020). LINC00467 Promotes Cell Proliferation and Metastasis by Binding with IGF2BP3 to Enhance the mRNA Stability of TRAF5 in Hepatocellular Carcinoma. J. Gene Med. 22 (3), e3134. 10.1002/jgm.3134 31656043

[B19] Li B. Mao R. Liu C. Zhang W. Tang Y. Guo Z. (2018). LncRNA FAL1 Promotes Cell Proliferation and Migration by Acting as a CeRNA of miR-1236 in Hepatocellular Carcinoma Cells. Life Sci. 197, 122–129. 10.1016/j.lfs.2018.02.006 29421439

[B20] Lim L. J. Wong S. Y. S. Huang F. Lim S. Chong S. S. Ooi L. L. (2019). Roles and Regulation of Long Noncoding RNAs in Hepatocellular Carcinoma. Cancer Res. 79 (20), 5131–5139. 10.1158/0008-5472.CAN-19-0255 31337653

[B21] Lobachevsky P. N. Bucknell N. W. Mason J. Russo D. Yin X. Selbie L. (2020). Monitoring DNA Damage and Repair in Peripheral Blood Mononuclear Cells of Lung Cancer Radiotherapy Patients. Cancers (Basel) 12 (9), 2517. 10.3390/cancers12092517 PMC7563254 32899789

[B22] Long J. Bai Y. Yang X. Lin J. Yang X. Wang D. (2019). Construction and Comprehensive Analysis of a ceRNA Network to Reveal Potential Prognostic Biomarkers for Hepatocellular Carcinoma. Cancer Cel Int 19, 90. 10.1186/s12935-019-0817-y PMC6458652 31007608

[B23] Ma H. Yuan L. Li W. Xu K. Yang L. (2018). The LncRNA H19/miR-193a-3p axis Modifies the Radio-Resistance and Chemotherapeutic Tolerance of Hepatocellular Carcinoma Cells by Targeting PSEN1. J. Cel Biochem 119 (10), 8325–8335. 10.1002/jcb.26883 29968942

[B24] Ma L. Xu A. Kang L. Cong R. Fan Z. Zhu X. (2021). LSD1 ‐Demethylated LINC01134 Confers Oxaliplatin Resistance through SP1 ‐Induced P62 Transcription in HCC. Hepatology 74 (6), 3213–3234. 10.1002/hep.32079 34322883

[B25] Mirman Z. de Lange T. (2020). 53BP1: a DSB Escort. Genes Dev. 34 (1-2), 7–23. 10.1101/gad.333237.119 31896689 PMC6938671

[B26] Reyes-Gibby C. C. Wang J. Silvas M. R. Yu R. Yeung S. C. Shete S. (2016). MAPK1/ERK2 as Novel Target Genes for Pain in Head and Neck Cancer Patients. BMC Genet. 17, 40. 10.1186/s12863-016-0348-7 26872611 PMC4752805

[B27] Rezatabar S. Karimian A. Rameshknia V. Parsian H. Majidinia M. Kopi T. A. (2019). RAS/MAPK Signaling Functions in Oxidative Stress, DNA Damage Response and Cancer Progression. J. Cell. Physiol. 10.1002/jcp.28334 30811039

[B28] Rim C. H. Lee H. Y. Kim J. S. Kim H. (2021). Radiofrequency Ablation and Stereotactic Body Radiotherapy for Hepatocellular Carcinoma: Should They Clash or Reconcile? Int. J. Radiat. Biol. 97 (2), 111–119. 10.1080/09553002.2021.1857453 33253598

[B29] Rong Z. Wang Z. Wang X. Qin C. Geng W. (2020). Molecular Interplay between Linc01134 and YY1 Dictates Hepatocellular Carcinoma Progression. J. Exp. Clin. Cancer Res. 39 (1), 61. 10.1186/s13046-020-01551-9 32272940 PMC7146959

[B30] Simon Y. Kessler S. M. Bohle R. M. Haybaeck J. Kiemer A. K. (2014). The Insulin-like Growth Factor 2 (IGF2) mRNA-Binding Protein p62/IGF2BP2-2 as a Promoter of NAFLD and HCC? Gut 63 (5), 861–863. 10.1136/gutjnl-2013-305736 24173291 PMC3995267

[B31] Song H. Liu Y. Li X. Chen S. Xie R. Chen D. (2020). Long Noncoding RNA CASC11 Promotes Hepatocarcinogenesis and HCC Progression through EIF4A3-Mediated E2F1 Activation. Clin. Transl Med. 10 (7), e220. 10.1002/ctm2.220 33252856 PMC7643871

[B32] Song J. Tian S. Yu L. Xing Y. Yang Q. Duan X. (2020). AC-caps: Attention Based Capsule Network for Predicting RBP Binding Sites of LncRNA. Interdiscip. Sci. 12 (4), 414–423. 10.1007/s12539-020-00379-3 32572768

[B33] Su M. Wang H. Wang W. Wang Y. Ouyang L. Pan C. (2018). LncRNAs in DNA Damage Response and Repair in Cancer Cells. Acta Biochim. Biophys. Sin (Shanghai) 50 (5), 433–439. 10.1093/abbs/gmy022 29554194

[B34] Sun J. Zhu Z. Li W. Shen M. Cao C. Sun Q. (2020). UBE2T-regulated H2AX Monoubiquitination Induces Hepatocellular Carcinoma Radioresistance by Facilitating CHK1 Activation. J. Exp. Clin. Cancer Res. 39 (1), 222. 10.1186/s13046-020-01734-4 33087136 PMC7576867

[B35] Taiana E. Favasuli V. Ronchetti D. Todoerti K. Pelizzoni F. Manzoni M. (2020). Long Non-coding RNA NEAT1 Targeting Impairs the DNA Repair Machinery and Triggers Anti-tumor Activity in Multiple Myeloma. Leukemia 34 (1), 234–244. 10.1038/s41375-019-0542-5 31427718

[B36] Tang Z. Y. (2000). Hepatocellular Carcinoma. J. Gastroenterol. Hepatol. 15 (Suppl. l), G1–G7. 10.1046/j.1440-1746.2000.02257.x 11100985

[B37] Wang D. F. Chen S. D. Zhu G. M. Gong W. D. (2018). Research Progress in Radiotherapy for Hepatocellular Carcinoma. Zhonghua Gan Zang Bing Za Zhi 26 (3), 238–240. 10.3760/cma.j.issn.1007-3418.2018.03.013 29804398 PMC12770651

[B38] Wang H. Ke J. Guo Q. Barnabo Nampoukime K. P. Yang P. Ma K. (2018). Long Non-coding RNA CRNDE Promotes the Proliferation, Migration and Invasion of Hepatocellular Carcinoma Cells through miR-217/MAPK1 axis. J. Cel Mol Med 22 (12), 5862–5876. 10.1111/jcmm.13856 PMC6237590 30246921

[B39] Wang Y. Wang Y. Luo W. Song X. Huang L. Xiao J. (2020). Roles of Long Non-coding RNAs and Emerging RNA-Binding Proteins in Innate Antiviral Responses. Theranostics 10 (20), 9407–9424. 10.7150/thno.48520 32802200 PMC7415804

[B40] Wen Z. Lian L. Ding H. Hu Y. Xiao Z. Xiong K. (2020). LncRNA ANCR Promotes Hepatocellular Carcinoma Metastasis through Upregulating HNRNPA1 Expression. RNA Biol. 17 (3), 381–394. 10.1080/15476286.2019.1708547 31868085 PMC6999620

[B41] Wu C. H. Chen C. Y. Yeh C. T. Lin K. H. (2020). Radiosensitization of Hepatocellular Carcinoma through Targeting Radio-Associated MicroRNA. Int. J. Mol. Sci. 21 (5). 10.3390/ijms21051859 PMC7084923 32182776

[B42] Wu M. Tong C. W. S. Yan W. To K. K. W. Cho W. C. S. (2019). The RNA Binding Protein HuR: A Promising Drug Target for Anticancer Therapy. Curr. Cancer Drug Targets 19 (5), 382–399. 10.2174/1568009618666181031145953 30381077

[B43] Wu X. Sui Z. Zhang H. Wang Y. Yu Z. (2020). Integrated Analysis of lncRNA-Mediated ceRNA Network in Lung Adenocarcinoma. Front. Oncol. 10, 554759. 10.3389/fonc.2020.554759 33042838 PMC7523091

[B44] Yang J. Li C. Li H. E C. (2019). LncRNA CACNA1G-AS1 Facilitates Hepatocellular Carcinoma Progression through the miR-2392/C1orf61 Pathway. J. Cel Physiol 234 (10), 18415–18422. 10.1002/jcp.28477 30908634

[B45] Yao Z. Zhang Y. Xu D. Zhou X. Peng P. Pan Z. (2019). Research Progress on Long Non-coding RNA and Radiotherapy. Med. Sci. Monit. 25, 5757–5770. 10.12659/MSM.915647 31375656 PMC6690404

[B46] Ye Y. Guo J. Xiao P. Ning J. Zhang R. Liu P. (2020). Macrophages-induced Long Noncoding RNA H19 Up-Regulation Triggers and Activates the miR-193b/MAPK1 axis and Promotes Cell Aggressiveness in Hepatocellular Carcinoma. Cancer Lett. 469, 310–322. 10.1016/j.canlet.2019.11.001 31705929

[B47] Yu Y. Feng M. (2018). Radiotherapy for Hepatocellular Carcinoma. Semin. Radiat. Oncol. 28 (4), 277–287. 10.1016/j.semradonc.2018.06.005 30309638

[B48] Zhang Y. He Q. Hu Z. Feng Y. Fan L. Tang Z. (2016). Long Noncoding RNA LINP1 Regulates Repair of DNA Double-Strand Breaks in Triple-Negative Breast Cancer. Nat. Struct. Mol. Biol. 23 (6), 522–530. 10.1038/nsmb.3211 27111890 PMC4927085

[B49] Zheng S. Guo Y. Dai L. Liang Z. Yang Q. Yi S. (2020). Long Intergenic Noncoding RNA01134 Accelerates Hepatocellular Carcinoma Progression by Sponging microRNA-4784 and Downregulating Structure Specific Recognition Protein 1. Bioengineered 11 (1), 1016–1026. 10.1080/21655979.2020.1818508 32970959 PMC8291876

[B50] Zhong J. H. Xiang X. Wang Y. Y. Liu X. Qi L. N. Luo C. P. (2020). The lncRNA SNHG16 Affects Prognosis in Hepatocellular Carcinoma by Regulating P62 Expression. J. Cel Physiol 235 (2), 1090–1102. 10.1002/jcp.29023 31256427

[B51] Zhou R. S. Zhang E. X. Sun Q. F. Ye Z. J. Liu J. W. Zhou D. H. (2019). Integrated Analysis of lncRNA-miRNA-mRNA ceRNA Network in Squamous Cell Carcinoma of Tongue. BMC cancer 19 (1), 779. 10.1186/s12885-019-5983-8 31391008 PMC6686570

