# Supplementary figures and images for: LncRNA LINC01134 Contributes to Radioresistance in Hepatocellular Carcinoma by Regulating DNA Damage Response via MAPK Signaling Pathway
Source: Front Pharmacol. 2022 Jan 31;12:791889. doi: 10.3389/fphar.2021.791889 (PMC8841450; doi:10.3389/fphar.2021.791889)

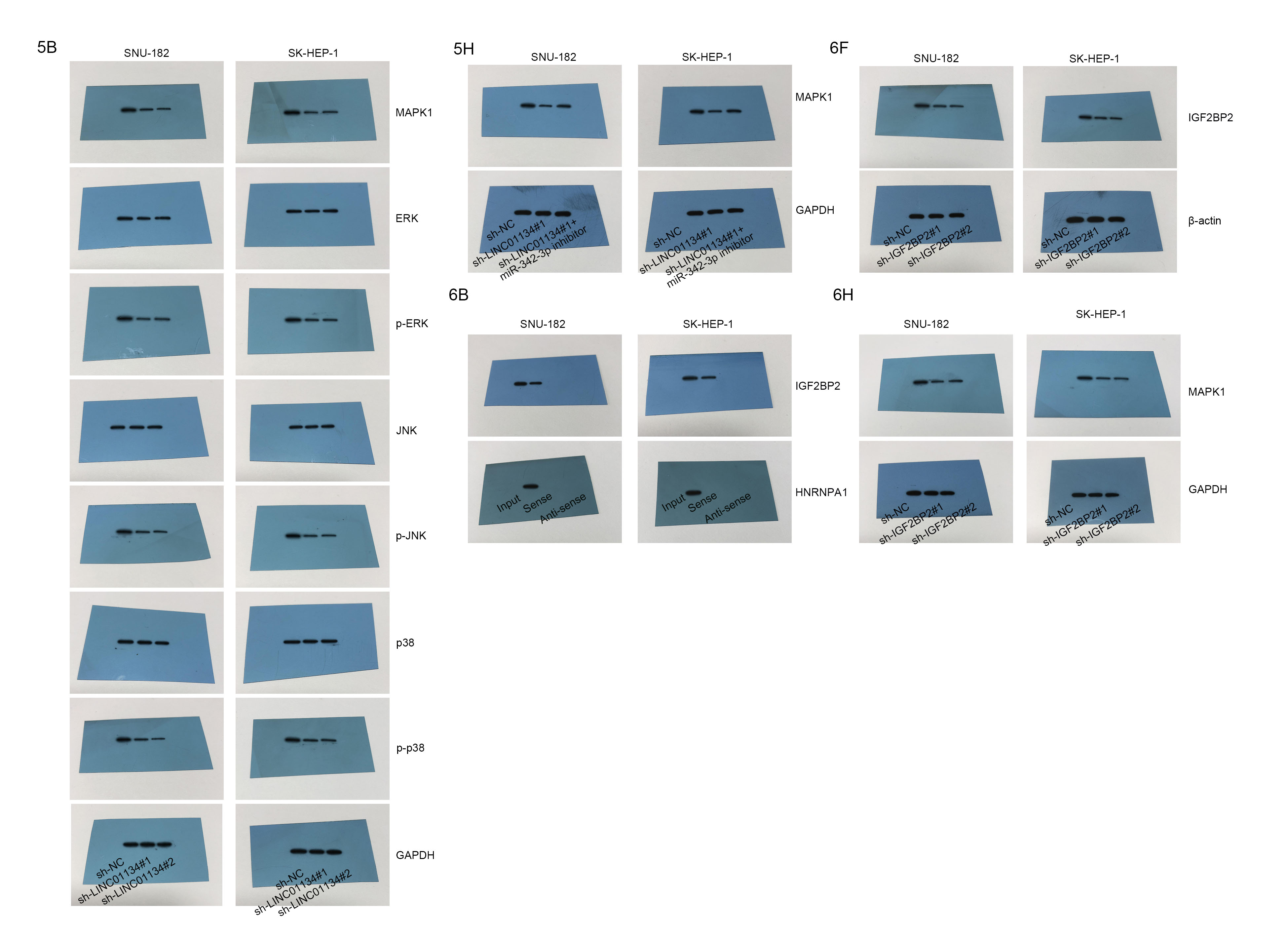

Supplement: Supplementary file 1 [file Image3.JPEG]

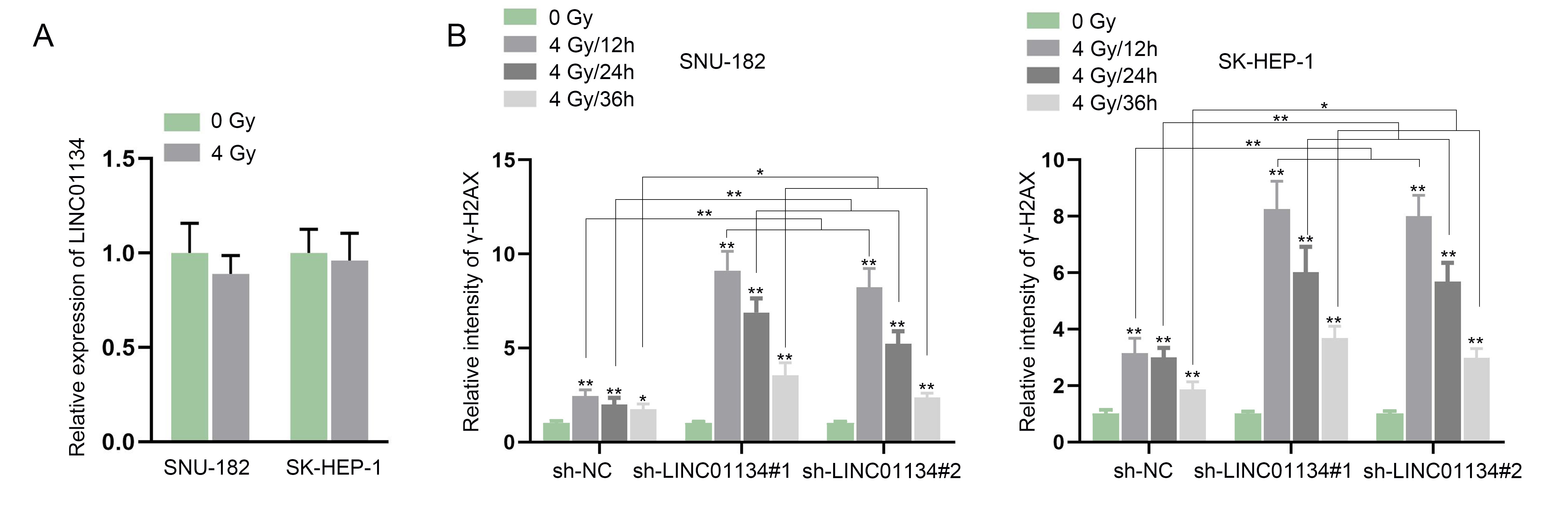

Supplement: Supplementary file 2 [file Image1.JPEG]

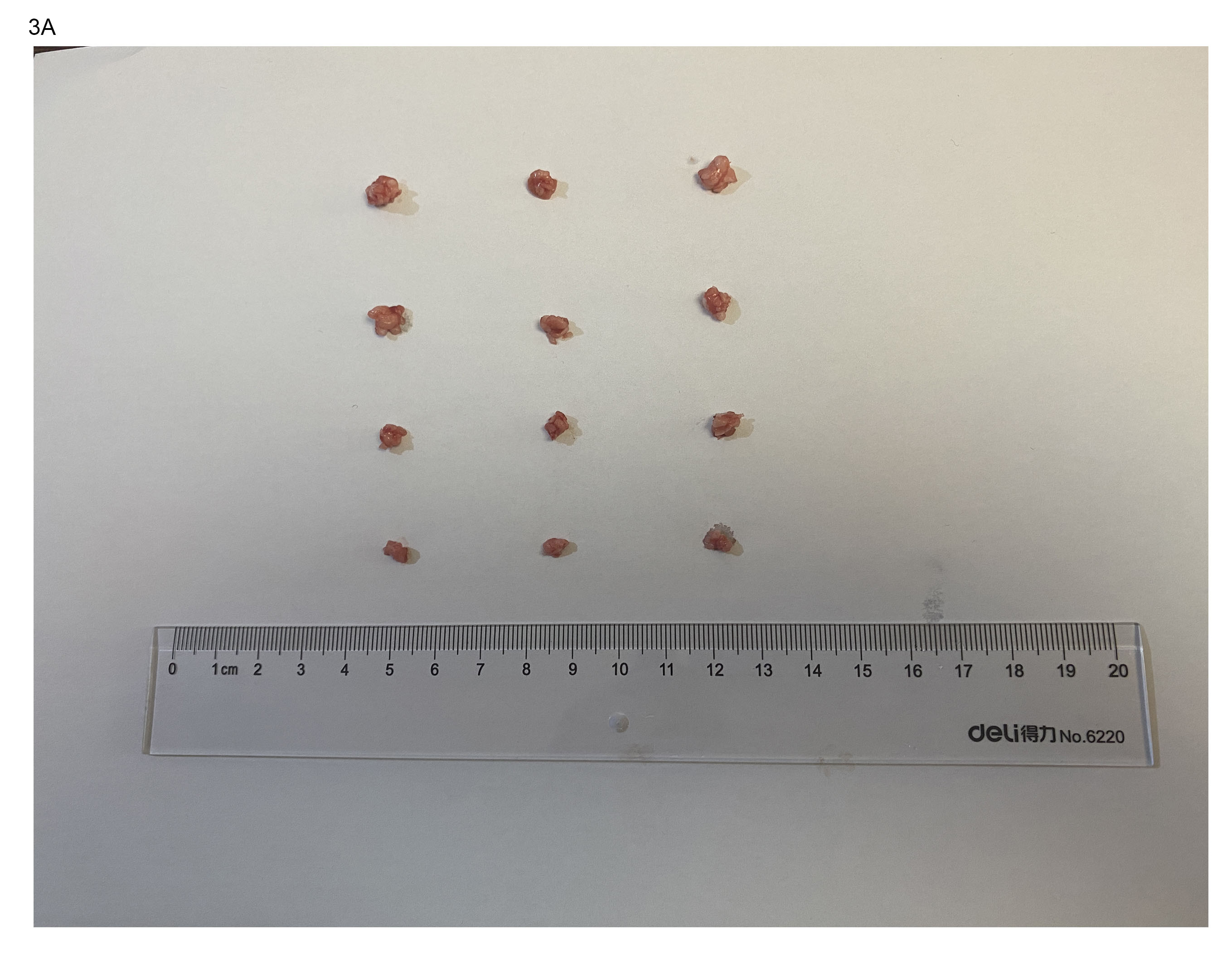

Supplement: Supplementary file 3 [file Image2.JPEG]
